# An Investigation of Factors Influencing the Postponement of the Use of Distributed Research Networks in South Korea: Web-Based Users’ Survey Study

**DOI:** 10.2196/40660

**Published:** 2023-04-12

**Authors:** Mi Jung Rho, Jihwan Park

**Affiliations:** 1 College of Health Science, Dankook University Cheonan-si Republic of Korea; 2 Department of Computer Education Dankook Liberal Art College Dankook University Cheonan-si, Chungcheongnam-do Republic of Korea

**Keywords:** distributed research networks, postponement, performance risk, workload

## Abstract

**Background:**

Distributed research networks (DRNs) offer researchers the advantage of using various big data sets that are difficult to access and use. In addition, since the data are not physically exposed to the outside, it is possible to conduct research using medical data safely without data exposure. However, researchers still have difficulties and are concerned about using DRNs. Few studies involving DRNs have been conducted from the user’s viewpoint. Therefore, it is necessary to look at DRNs from the researcher’s point of view and find ways to facilitate the active use of DRNs.

**Objective:**

This study aimed to identify the factors that made researchers hesitate to use DRNs and to derive a method to facilitate active DRN use.

**Methods:**

We conducted a web-based survey of people working in the medical fields, such as hospitals and universities. We used 131 respondents’ data from a survey from December 6 to 17, 2021. We conducted multiple regression analyses to determine the factors affecting the postponement of using DRNs. In addition, 2 independent sample *t* tests were conducted to analyze the difference between the 2 groups according to the following factors: organization, gender, experience with DRNs, length of the research career, position, and age.

**Results:**

Performance risk (*t*_5_=2.725, *P*=.007*)* and workload from DRNs (*t*_5_=3.543, *P*=.001) were significantly associated with users’ postponement of DRN use. Researchers working at hospitals were found to feel more burdened by DRN use than researchers working at universities (*t*_129_=1.975, *P*=.05). It was also found that women perceived a higher privacy risk of DRNs than men (*t*_129_=–2.303, *P*=.02) and that those who had experience using DRNs delayed their use less than those without experience (*t*_129_=–4.215, *P*<.001).

**Conclusions:**

It is necessary to simplify the research and approval processes to reduce the performance risk and workload of research using DRNs. To optimize the process, DRN providers should develop a way to improve users’ experiences. More user-friendly functionalities should be developed from the researcher's point of view. It is necessary to continuously promote effective functionalities for DRNs to reduce concerns about privacy risks. This study identified the concerns of DRN users in terms of DRN use and suggested ways to actively use DRNs. The derived results can be reflected in planning and developing DRNs. Our research will be helpful to prepare an activation plan for DRNs.

## Introduction

Distributed research networks (DRNs) are useful platforms for multi-institutional, global research and public health surveillance [1-3]. DRNs offer researchers the advantage of using various big data sets that are otherwise difficult to access and use.

Various types of DRNs, such as PCORnet [4,5], BBMRI-ERIC [6,7], and ELIXIR [8,9], have been used to access and use clinical data from various institutions. Research results for overcoming diseases using DRNs are being produced in quick succession [5,10-12]. In South Korea, various DRNs exist and are being used with national support, such as health care DRNs from the Korea Health Industry Development Institute and the medical record observation and assessment for the drug safety project [13,14].

Many papers have been published on clinical results using DRNs, governance, and data quality; there have also been studies related to policy or legislation and development direction [15-18]. However, it is still difficult for researchers to use DRNs. Few studies involving DRNs have been conducted from the user’s viewpoint. Therefore, it is necessary to look at DRNs from the researcher’s point of view and find ways to actively use DRNs.

Many previous studies have considered positive factors regarding the performance and effectiveness of DRNs. In 2009, national DRNs were said to be effective and were believed to offer superior quality for medical best practices [19]. In 2010, another study on distributed data networks for multi-institute electronic health data pointed out several factors relevant to using data from multiple organizations, such as the effectiveness of research, the safety of medical products, and quality [20]. Three years later, another study mentioned that the DRN analysis result-sharing method is effective without sharing identifiable data [21]. However, despite the benefits of DRNs, researchers are hesitant to use them. For this reason, we aimed to determine the factors that influence the user’s postponement of DRN use.

To encourage the use of DRNs, it is necessary to identify the main factors affecting their use postponement. We focused on the negative factors affecting the active use of DRNs. Postponing use of DRNs is due to the delay and concerns about using users’ DRNs. Observable issues with DRN use postponement include responsibilities [22], workload [23,24], and perceived risks [25,26]. Previous research has explained the responsibilities associated with some information from medical organizations [22]. Some research has mentioned why people resist new systems. One reason is that they worry about their workload; for instance, they need time to determine whether the current system is the best option [23,24]. A postponement tendency can be derived from the findings of these previous works [22-24] about resistance to a new system. Perceived risks include 3 factors: financial, performance, and privacy risks. For instance, purchasing an expensive system increases financial risk and decreases cost performance. Regarding privacy risk, installing a new system will create another management point that will require privacy management. Here, we aimed to examine the main factors affecting the postponement of the use of DRNs.

## Methods

### Instrument Development

In this study, we focused on factors influencing users’ postponement of the use of DRNs. DRNs are a new type of information technology used in health care research. Studies have been conducted on users’ perceptions of new information technology.

A previous study used 2 models, namely the technology acceptance model and the theory of planned behavior, to define influencing factors for the adoption of information systems, such as financial, performance, and privacy risks [25]. In 1972, perceived risk components had already been defined, including functional, psychological, and financial risks [26]. Furthermore, a study has been conducted on resistance to new information technology, responsibilities, and workload when adopting a new system [23]. Another study also mentioned resistance when implementing a medical management information system and found that a new medical information system creates more responsibilities and a heavier workload for doctors [24]. Thus, through a literature review, we developed a questionnaire to investigate the factors influencing the postponement of the use of DRNs. Based on previous studies, we considered perceived risks to encompass financial, performance, and privacy risks in users’ postponement of DRNs [25].

Based on the existing literature, we modified and defined the factors for innovation resistance to suit this research purpose (Table 1). Financial risk refers to the degree to which users are concerned that DRNs may not be worth the price paid. The price is paid by researchers for using the analysis results from the data of DRNs. This is why we have some questionnaires on financial risk. Performance risk refers to the degree to which users perceive that the performance of DRNs will not be useful in their research. Privacy risk refers to the degree to which there is concern over the possibility of excessive collection or leakage of medical data when using DRNs. In other words, privacy risk is about the trust needed to use the system. Responsibilities refer to the degree of concern about the user’s responsibilities associated with using DRNs. Workload refers to the degree of concern about increased workload because of the use of DRNs. Lastly, postponement refers to the user’s intention to postpone the decision to use DRNs.

**Table 1:** Definition of variables.

Variable			Definition		References
**Perceived risks**					
	Financial risk	The degree to which users are concerned that the DRNs^a^ may not be worth the price paid		[19,23]	
	Performance risk	The degree to which users perceive that the performance of DRNs will not be very useful in research		[19,23]	
	Privacy risk	Concern over the possibility of excessive collection or leakage of medical data when using DRNs		[19,23]	
Responsibilities			The degree of concern about the responsibilities associated with using DRNs		[2,8,11]
Workload			The degree of concern about increased workload because of the use of DRNs		[8,11]
Postponement			The user’s intention to postpone the decision to use the DRN		[8,11]

^a^DRN: distributed research network.

### Ethics Approval

This study was performed in accordance with the Declaration of Helsinki and was approved by the institutional review board of the Dankook University of Korea (DKU2021-08-013-003).

### Informed Consent

An explanatory statement about the study was presented to the participants, and informed consent was obtained for data collection and analysis. All collected personal information of the participants was deleted, and all participants’ personal information was anonymized and used for analysis. We provided coffee vouchers to participants who completed the survey.

### Statistical Analysis

#### Analysis

We used SPSS (version 26.0; SPSS Inc) to perform the usability evaluation analysis. We conducted multiple regression analyses to determine the factors affecting postponement. Furthermore, 2 independent sample *t* tests [27] were conducted to analyze the difference between the 2 groups according to the following factors: organization (hospital vs university), sex (male vs female), DRN experience (yes or no), length of research career (<5 vs ≥5), position (professor, researcher, doctor, nurse, or pharmacist vs student, including master’s and doctoral programs, etc), and age (20s or 30s vs over 40 years).

#### Sample and Data Collection

This study focused on the users who use DRNs for research purposes. We conducted a web-based survey of people working in medical fields, such as hospitals and universities. Before the survey, we delivered a DRN-related explanation to participants to ensure that they understood. The delivered explanation was about its concept, to ensure participants understood its strengths and opportunities. Data were collected using the snowball method [28] because few researchers have used DRNs. The snowball method involves gathering many participants from a small initial group, propagating to the others, and creating large groups through snowball growth. In this study, the first 5 researchers disseminated the survey link, and each of the 5 initial recipients passed the survey on to another person, and so on. Surveys were administered from December 6 to 17, 2021. The final sample includes data from 131 respondents.

## Results

### Demographic Characteristics

Within the sample, the sex ratio was fairly evenly balanced (Table 2). Of the respondents, 38.9% (51/131) were in their 30s, and 27.5% (36/131) were in their 40s. Additionally, 63.4% (83/131) of the respondents worked at organizations located in Seoul. About 51.1% (67/131) worked at universities, and 48.9% (64/131) worked in hospitals. Of the respondents, 29% (38/131) were in a research position, followed by professors, who accounted for 26.7% (35/131). Those with less than 5 years of research experience were the most numerous, at 40.5% (53/131), and 33.6% (44/131) had more than 11 years of research experience. The main research field was medicine, which accounted for 48.1% (63/131) of the total. Finally, only 11.5% (15/131) of the respondents had experience with DRNs.

**Table 2:** Demographic characteristics (N=131).

Variables		Participants, n (%)
**Sex**		
	Male	64 (48.9)
	Female	67 (51.1)
**Age**		
	20-29 years	15 (11.5)
	30-39 years	51 (38.9)
	40-49 years	36 (27.5)
	≥50 years	29 (22.1)
**Location**		
	Seoul	83 (63.4)
	Busan	5 (3.8)
	Daegu Metropolitan City	4 (3.1)
	Incheon Metropolitan City	5 (3.8)
	Gwangju	2 (1.5)
	Daejeon	3 (2.3)
	Ulsan Metropolitan City	1 (0.8)
	Gyeonggi-do	18 (13.7)
	Gangwon-do	4 (3.1)
	Chung-cheong bukdo	2 (1.5)
	Chungcheongnam-do	1 (0.8)
	Jeollabuk-do	2 (1.5)
	Gyeongsangbuk-do	1 (0.8)
**Organization**		
	Hospital	64 (48.9)
	University	67 (51.1)
**Position**		
	Professor	35 (26.7)
	Researcher	38 (29.0)
	Doctor, nurse, or pharmacist	23 (17.6)
	Students (including those in masters and doctoral programs)	25 (19.1)
	Others	10 (7.6)
**Length of research career**		
	<5 years	53 (40.5)
	5-10 years	34 (26.0)
	≥11 years	44 (33.6)
**Research field**		
	Medicine	63 (48.1)
	Natural science	31 (23.7)
	Engineering	10 (7.6)
	Convergence department	27 (20.6)
**Experience with distributed research networks**		
	Yes	15 (11.5)
	No	116 (88.5)

### Reliability and Validity

We performed a principal component analysis with varimax rotation to determine the quality factor. All factors emerged with no cross-construct loadings above 0.60 (Table 3). The results confirmed the existence of all factors with eigenvalues greater than 1.0, which accounted for 74.07% of the total variance. The communality ranged from 0.604 to 0.846. All items satisfied a 0.50 threshold. Cronbach α for all factors was found to be in the reliable range, and the values for all factors were greater than 0.60. Values ranged from 0.646 to 0.910 (postponement: 0.910, privacy risk: 0.896, responsibilities: 0.861, performance risk: 0.766, financial risk: 0.646, and workload: 0.722).

**Table 3:** Loadings, cross-loadings, and reliability.

Factors and items			Component													Cronbach α			Communality
		1		2		3		4		5		6							
**Postponement (Post)**															.910				
	Post2	.878		.095		–.059		.125		–.031		.151					.823		
	Post3	.845		.036		–.093		.120		.066		.262					.811		
	Post1	.836		.162		.003		.250		.062		.087					.800		
	Post4	.830		.202		–.018		.116		.126		.089					.767		
**Privacy risk (PR)**															.896				
	PR3	.014		.839		–.065		.124		.080		.218					.777		
	PR1	.164		.831		–.119		.123		.145		.106					.779		
	PR4	.217		.817		–.220		.143		.016		.201					.824		
	PR5	.154		.757		–.097		.327		.075		.070					.724		
**Responsibilities (Re)**															.861				
	Re3	.035		–.023		.889		–.025		–.119		–.048					.810		
	Re2	–.055		–.134		.855		.031		–.011		–.092					.761		
	Re4	–.122		–.243		.851		–.083		–.044		.094					.817		
**Performance risk (PR)**															.766				
	PR2	.165		.255		.058		.863		.063		.050					.846		
	PR4	.277		.268		–.045		.733		–.018		.093					.697		
	PR1	.146		.088		–.098		.658		.259		.294					.625		
**Financial risk (FR)**															.646				
	FR2	.157		.136		–.179		–.006		.754		.130					.661		
	FR3	–.060		.176		.200		.062		.748		–.015					.638		
	FR4	.071		–.057		–.196		.148		.730		.052					.604		
**Workload (WL)**															.722				
	WL3	.230		.154		.008		–.011		.023		.821					.751		
	WL4	.304		.223		–.120		.355		.010		.637					.688		
	WL2	.115		.299		.014		.294		.223		.610					.612		
Eigenvalue			3.307		3.151		2.475		2.225		1.857		1.799		—^a^			—	
Variance (%)			16.534		15.756		12.374		11.127		9.284		8.996		—			—	
Cumulative percentage (%)			16.534		32.289		44.664		55.791		65.075		74.070		—			—	

^a^Not available.

### Negative Factors Affecting the User’s Postponement of DRN Use

The results identified factors that impact the user’s postponement of DRN use. The results indicated that both independent variables, that is, performance risk and workload, significantly contributed to the postponement of DRNs use (*F*_5_=10.759, *P*<.001). The coefficient of determination (*R*^2^) for this model was 0.301, indicating that 30.1% of the variation in the postponement of DRNs use is explained by the 2 independent variables.

Table 4 shows that performance risk (*t*_5_=2.725, *P*=.007) and workload (*t*_5_=3.543, *P=*.001) were significantly associated with the user’s postponement of DRN use. However, privacy risk, responsibilities, and financial risk had no substantial relationship with the user’s postponement of DRN use. According to the standardized coefficients representing the relative contributions of the independent variables, the workload had a substantial effect on the user’s postponement of DRN use (β=.328) while performance risk (β=.252) influenced the user’s postponement of DRN use.

**Table 4:** Multiple regression analysis results for users’ postponement of DRN use.

Independent variables	Nonstandardized coefficients			Standardized coefficients		*t* value		*P* value		Collinearity	
	β	SE	β						Tolerance		VIF^a^
Constant	1.449	.559	—^b^		2.592		.01		—		—
Privacy risk	.051	.095	.051		0.540		.59		.623		1.604
Responsibilities	–.048	.080	–.047		–0.600		.55		.901		1.110
Performance risk	.313	.115	.252		2.725		.007^c^		.652		1.534
Financial risk	.007	.120	.005		0.057		.96		.898		1.113
Workload	.375	.106	.328		3.543		.001^c^		.651		1.536

^a^VIF: variance inflation factor.

^b^Not applicable.

^c^*t*_0.01_=2.576, *R*^2^ (adjusted *R*^2^)=0.301 (0.273), *F* change=10.759, significance for *F* change=0.000.

### Differences Between the Groups in Perception

We conducted 2 independent sample *t* tests to analyze the perceived differences between the 2 groups according to the following factors: organization, sex, experience with DRNs, length of the research career, position, and age (Table 5). The dependent variables were privacy risk, responsibilities, performance risk, financial risk, workload, and the user’s postponement of DRN use. We excluded results that were not statistically significant.

Researchers working at hospitals were found to feel more burdened than researchers working at universities (*t*_129_=1.975, *P=.*05). The results showed that women perceived a higher privacy risk than men (*t*_129_=–2.303, *P=.*02) and that those who had experience using DRNs delayed using them less than those without experience (*t*_129_=–4.215, *P*<.001). However, there was no statistically significant difference in perceptions of the 6 variables in terms of research experience or classification by occupational group or by age.

**Figure 1:**
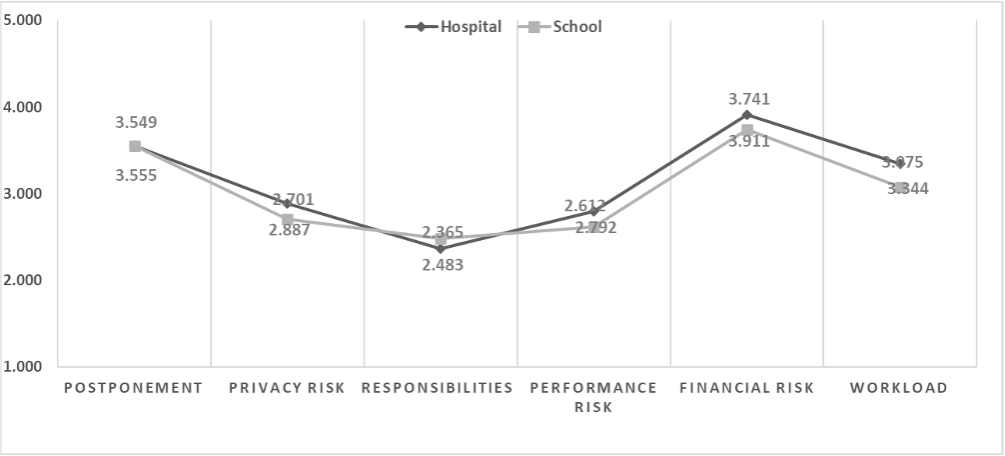
Differences in perception according to work organization.

Although statistical significance was not found for the other factors, we obtained interesting survey mean scores from the participants. For instance, both groups of researchers at hospitals and universities indicated low recognition of privacy risks, responsibilities, and performance risks when using DRNs. However, they worried about the workload associated with DRNs as the new system (Table S1 in Multimedia Appendix 1 and Figure 1).

**Figure 2:**
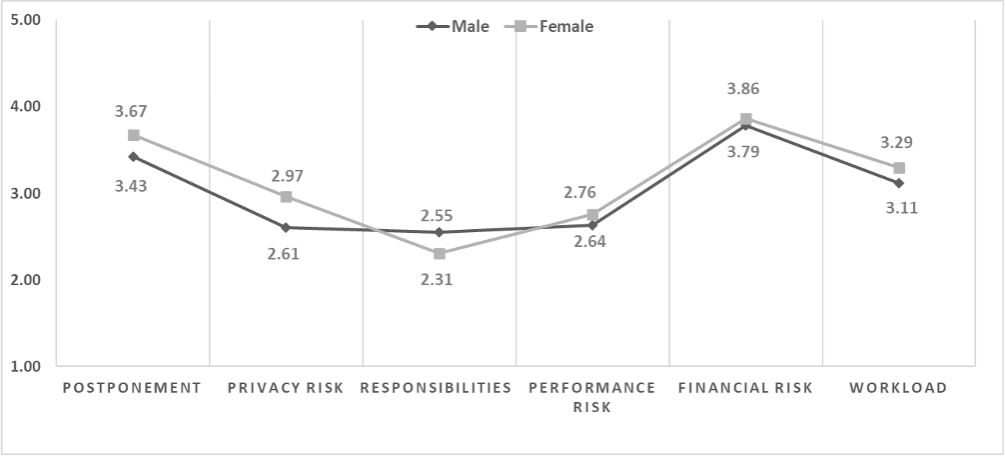
Differences in perception according to sex.

The male and female groups indicated low recognition of privacy risks, responsibilities, and performance risks. They were worried about financial risk and workload from DRNs (see Table S2 in Multimedia Appendix 1 and Figure 2). Although there was no statistically significant difference, female participants were more likely to delay using DRNs than male participants.

**Figure 3:**
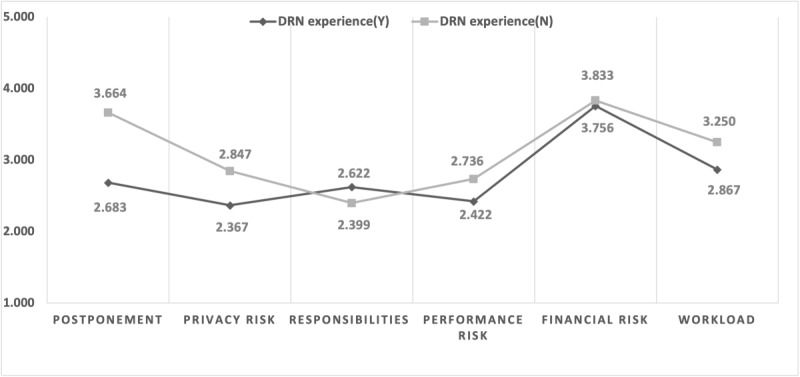
Differences in perception according to experience with distributed research networks (DRNs).

Another group with experience with DRNs indicated low recognition of privacy risks, responsibilities, and performance risks. They were worried about financial risk and workload (see Table S3 in Multimedia Appendix 1 and Figure 3). Although there was no statistically significant difference, those with DRN experience felt that they had more responsibilities than those without experience.

**Table 5:** Results of 2 independent sample t tests.

Dependent variables and group		N	Mean (SD)	*t* value (*df*)	*P* value
**Workload**				1.975 (129)	.05^a^
	Hospital	64	3.344 (0.786)		
	University	67	3.075 (0.774)		
**Privacy risk**				–2.304 (129)	.02^a^
	Male	64	2.609 (0.935)		
	Female	67	2.966 (0.838)		
**Postponement**				–4.215 (129)	<.001^b^
	Experience with DRNs^c^ (yes)	15	2.683 (1.112)		
	Experience with DRNs (no)	116	3.664 (0.810)		

^a^*t*_0.05_=1.960.

^b^*t*_0.001_=3.291.

^c^DRN: distributed research network.

Finally, the other groups that differed according to the length of the research career, position, and age showed similar results. Ultimately, the majority of participants were worried about their financial risk and workload from DRNs (Multimedia Appendices 2-4).

## Discussion

### Principal Findings

The following conclusions were drawn based on this study’s results.

The first interesting result is that among financial risk [25,26], performance risk [25,26], privacy risk [25,26], responsibilities [22-24], and workload [23,24], only performance risk and workload from DRNs were found to affect users’ postponement of DRN use. In other words, delays in DRN use increase because the performance of DRN is perceived to be poor. The more potential users think their workload will increase, the longer the delay in using DRNs. Among the examined factors, workload from DRNs had the greatest influence on users’ postponement of DRN use. Regarding the electronic health records system, it is also reported that improvements in usability are related to physicians’ perceptions of workload and system performance [29]. Researchers have confirmed that they do not wish to increase their research workload by using DRNs. Therefore, it is necessary to simplify the research and approval processes. Avoiding the creation of unnecessary workflows is desirable.

Regarding DRNs, researchers are interested in determining whether the system is well-suited to their research and whether it will provide immediate support functionally and conveniently. The system seems to be developing from the perspective of the platform provider, in that data are shared and used in the construction of DRNs. It should be remembered that even though DRNs provide a variety of large, multiorganizational data, they are ultimately meaningless unless users actively use them. More user-friendly functionalities should be developed from the researcher’s point of view.

As a result of analyzing the differences in perception among the 6 groups, the respondents’ perception of postponement was negative overall. Their perceptions of financial risk and workload were the poorest among the 5 variables (ie, financial risk, performance risk, privacy risk, responsibilities, and workload). Relatively, the response values for privacy risk, responsibility, and performance risk were moderate.

Prevalent postponement of DRN use is a phenomenon that DRN providers should monitor. Drastic delays in DRN use have been noted despite the great advantage it offers researchers in terms of accessing data that would otherwise be difficult for individual researchers to access. An approach from the researchers’ perspective that can lower awareness of the postponement of DRN use is urgently needed.

It is also worth noting that financial risk and workload were perceived to be high. To encourage the use of DRNs, it is desirable to incentivize organizations that provide data. Furthermore, it is necessary to reduce cost concerns and enable researchers who do not have funds to use DRNs. As mentioned earlier, avoiding increasing the workload on researchers because of the use of DRNs is crucial. The risk of an increased workload appears to be the most important factor in the quest to encourage the use of DRNs.

However, when participants were grouped according to research experience, occupational group, and age and analyzed, there were no statistically significant differences between the 2 groups with regard to the 6 variables: financial risk, performance risk, privacy risk, responsibilities, workload, and postponement of DRN use. In other words, perceptions of DRNs were found to be similar across the health care field, regardless of professionals’ study period, age, or job group.

Next, researchers working at hospitals were concerned that DRNs would further increase their research workload. For researchers employed at hospitals, the additional workload associated with DRNs is daunting because it requires the completion of other components of their overall workload. In health care, studies on burnout and workload are numerous and ongoing [30-32]. Therefore, despite the merits of the data DRNs deliver, there may be a burden associated with DRN use. For this reason, it is desirable to minimize the unnecessary processes that arise during the research process. To optimize the process, DRN providers should develop a way to improve users’ experiences. It is necessary to continuously promote effective functionalities for DRNs.

### Comparison With Prior Work

Studies have shown that female researchers are less tech-friendly and less active in their use of technology than male researchers [33,34]. However, in this study, no sex differences were found in perceptions of the postponement of DRN use, in keeping with the results of a study that showed that the more experience women gained with technology, the narrower the sex gap in technology use became [35].

It was also found that female researchers were more concerned about the privacy issues of DRNs than male researchers. In a previous study, female users reported lower information security and security behaviors than male users [36]. Thus, female users are more likely to exhibit poor information security behavior. Our results differ from those of this study, likely because their respondent group was a group of experts. Privacy issues should be considered when designing DRNs. Female researchers’ greater concern about privacy risks can be overcome through an appropriate publicity campaign. In this study, we suggest that the campaign that helps all the users, whether they are men or women, can understand the safety of the DRNs, which do not allow the delivery of the raw data outside but provide safe analysis results.

Furthermore, persons with experience using DRNs responded positively to delays in using them. Experience with DRNs is therefore highly recommended since experienced researchers are less negative about delayed use. Regarding mobile health monitoring services, there exists a study in which users perceived the benefits of the service as offering an advantage over nonusers [37]. They also reported less concern about the risks of using the service. Thus, efforts should be made to continuously promote the advantages of DRNs to encourage researchers to use them.

### Limitations

Despite these substantial results, this study had several limitations. First, our data are affected by the disadvantage of a small proportion of people who experienced DRNs versus those who had not among the total sample of 131. Future studies using more data from experienced DRNs users are needed. Second, although DRNs exist in South Korea, the majority of researchers have not yet actively used them. It seems desirable to approach the same research topic after DRN use increases. Third, this study targeted Korean researchers in the health care industry. Therefore, the results are limited in terms of representing various DRN users abroad, such as users of PCORnet, BBMRI-ERIC, and ELIXIR. Lastly, this study focused DRNs user-side; future studies may use the topic of the administration of DRNs.

### Conclusions

Despite these limitations, our results provide insights that can contribute to overcoming the delayed use of DRNs. Many existing studies have focused on the intention to use new technology in the health care industry [38-40]. However, we focused specifically on the postponement of DRN use. Furthermore, responsibility factors were selected and analyzed in terms of innovation resistance [23,24]; that is, when a new system is implemented within a medical organization, medical workers resist or postpone using the system. Previous studies and projects in Korea have promoted the advantages of DRN use [8,12-14], but despite awareness of these merits, many organizations have still postponed the use of DRNs. Since this study focused on the user’s point of view regarding the factors influencing the postponement of DRN use, these research findings are valuable for encouraging its use. Our research will be helpful in preparing an activation plan that can be used in various ways. It is meaningful that we looked at the postponement of DRN use from the viewpoint of resistance to innovation.

## Appendix

Multimedia Appendix 1 Results of the independent sample *t* tests.

Multimedia Appendix 2 Differences in perception according to the length of the research career.

Multimedia Appendix 3 Differences in perception according to the job or position.

Multimedia Appendix 4 Differences in perception according to the age group.

## Abbreviations

DRN: distributed research network

## Data Availability

The data sets used and analyzed during this study are not publicly available due to the lack of a web link for a repository but are available from the corresponding author on request.
