# Supplementary material for: An Investigation of Factors Influencing the Postponement of the Use of Distributed Research Networks in South Korea: Web-Based Users’ Survey Study
Source: JMIR Form Res. 2023 Apr 12;7:e40660. doi: 10.2196/40660 (PMC10134027; doi:10.2196/40660)
Supplement: Multimedia Appendix 1 [file formative_v7i1e40660_app1.docx]

Multimedia Appendix 1

**Table S1.** Results of 2 independent sample *t* tests according to organization.

| Dependent variables | Organization | N | Mean | SD | t-value | Sig. |
| --- | --- | --- | --- | --- | --- | --- |
| Postponement | Hospital | 64 | 3.555 | .917 | .039 | .969 |
|  | University | 67 | 3.549 | .892 |  |  |
| Privacy risk | Hospital | 64 | 2.887 | .875 | 1.177 | .241 |
|  | University | 67 | 2.701 | .923 |  |  |
| Responsibilities | Hospital | 64 | 2.365 | .890 | -.756 | .451 |
|  | University | 67 | 2.483 | .896 |  |  |
| Performance risk | Hospital | 64 | 2.792 | .745 | 1.423 | .157 |
|  | University | 67 | 2.612 | .700 |  |  |
| Financial risk | Hospital | 64 | 3.911 | .561 | 1.658 | .100 |
|  | University | 67 | 3.741 | .611 |  |  |
| Workload | Hospital | 64 | 3.344 | .786 | 1.975 | 0.05* |
|  | University | 67 | 3.075 | .774 |  |  |

*t*_0.05_=1.960.

**Table S2.** Results of 2 independent sample *t* tests according to sex.

| Dependent variables | Sex | N | Mean | SD | t-value | Sig. |
| --- | --- | --- | --- | --- | --- | --- |
| Postponement | Male | 64 | 3.426 | .960 | -1.570 | .119 |
|  | Female | 67 | 3.672 | .829 |  |  |
| Privacy risk | Male | 64 | 2.609 | .935 | -2.303 | .023* |
|  | Female | 67 | 2.966 | .838 |  |  |
| Responsibilities | Male | 64 | 2.547 | .880 | 1.538 | .127 |
|  | Female | 67 | 2.308 | .894 |  |  |
| Performance risk | Male | 64 | 2.635 | .762 | -.992 | .323 |
|  | Female | 67 | 2.761 | .689 |  |  |
| Financial risk | Male | 64 | 3.786 | .618 | -.717 | .474 |
|  | Female | 67 | 3.861 | .566 |  |  |
| Workload | Male | 64 | 3.115 | .777 | -1.302 | 0.195 |
|  | Female | 67 | 3.294 | .795 |  |  |

*t*_0.05_=1.960.

**Table S3.** Results of 2 independent sample *t* tests according to experience with DRNs.

| Dependent variables | Experience with DRNs | N | Mean | SD | t-value | Sig. |
| --- | --- | --- | --- | --- | --- | --- |
| Postponement | Y | 15 | 2.683 | 1.112 | -4.215 | .000*** |
|  | N | 116 | 3.664 | .810 |  |  |
| Privacy risk | Y | 15 | 2.367 | .737 | -1.963 | .052 |
|  | N | 116 | 2.847 | .909 |  |  |
| Responsibilities | Y | 15 | 2.622 | 1.061 | .910 | .364 |
|  | N | 116 | 2.399 | .869 |  |  |
| Performance risk | Y | 15 | 2.422 | .947 | -1.584 | .116 |
|  | N | 116 | 2.736 | .689 |  |  |
| Financial risk | Y | 15 | 3.756 | .479 | -.478 | .633 |
|  | N | 116 | 3.833 | .605 |  |  |
| Workload | Y | 15 | 2.867 | 1.090 | -1.787 | 0.076 |
|  | N | 116 | 3.250 | .735 |  |  |

****t*_0.001_=3.291.

**Table S4.** Results of 2 independent sample *t* tests according to the length of career.

| Dependent variables | Length of research career | N | Mean | SD | t-value | Sig. |
| --- | --- | --- | --- | --- | --- | --- |
| Postponement | 5 years or less | 53 | 3.623 | .891 | .744 | .458 |
|  | More than 6 years | 78 | 3.503 | .909 |  |  |
| Privacy risk | 5 years or less | 53 | 2.901 | .899 | 1.142 | .256 |
|  | More than 6 years | 78 | 2.718 | .901 |  |  |
| Responsibilities | 5 years or less | 53 | 2.396 | .803 | -.303 | .763 |
|  | More than 6 years | 78 | 2.444 | .951 |  |  |
| Performance risk | 5 years or less | 53 | 2.686 | .649 | -.184 | .854 |
|  | More than 6 years | 78 | 2.709 | .777 |  |  |
| Financial risk | 5 years or less | 53 | 3.811 | .625 | -.208 | .835 |
|  | More than 6 years | 78 | 3.833 | .570 |  |  |
| Workload | 5 years or less | 53 | 3.296 | .712 | 1.072 | .286 |
|  | More than 6 years | 78 | 3.145 | .835 |  |  |

**Table S5**. Results of 2 independent sample *t* tests according to the job or position.

| Dependent variables | Position | N | Mean | SD | t-value | Sig. |
| --- | --- | --- | --- | --- | --- | --- |
| Postponement | Professor/Doctor/Nurse/Pharmacist | 58 | 3.534 | .892 | -.192 | .848 |
|  | Researcher/Students and Etc. | 73 | 3.565 | .914 |  |  |
| Privacy risk | Professor/Doctor/Nurse/Pharmacist | 58 | 2.737 | .934 | -.620 | .536 |
|  | Researcher/Students and Etc. | 73 | 2.836 | .879 |  |  |
| Responsibilities | Professor/Doctor/Nurse/Pharmacist | 58 | 2.374 | .924 | -.586 | .559 |
|  | Researcher/Students and Etc. | 73 | 2.466 | .869 |  |  |
| Performance risk | Professor/Doctor/Nurse/Pharmacist | 58 | 2.649 | .773 | -.706 | .481 |
|  | Researcher/Students and Etc. | 73 | 2.740 | .688 |  |  |
| Financial risk | Professor/Doctor/Nurse/Pharmacist | 58 | 3.741 | .555 | -1.440 | .152 |
|  | Researcher/Students and etc. | 73 | 3.890 | .614 |  |  |
| Workload | Professor/Doctor/Nurse/Pharmacist | 58 | 3.184 | .773 | -.286 | .775 |
|  | Researcher/Students and Etc. | 73 | 3.224 | .805 |  |  |

**Table S6**. Results of 2 independent sample *t* tests according to the age group.

| **Dependent variables** | **Age group** | ***N*** | **Mean** | **SD** | ***t*-value** | **Sig.** |
| --- | --- | --- | --- | --- | --- | --- |
| Postponement | 20s and 30s | 66 | 3.527 | .931 | -.319 | .750 |
|  | Over 40 | 65 | 3.577 | .875 |  |  |
| Privacy risk | 20s and 30s | 66 | 2.708 | .840 | -1.071 | .286 |
|  | Over 40 | 65 | 2.877 | .959 |  |  |
| Responsibilities | 20s and 30s | 66 | 2.429 | .808 | .056 | .955 |
|  | Over 40 | 65 | 2.421 | .976 |  |  |
| Performance risk | 20s and 30s | 66 | 2.672 | .727 | -.444 | .658 |
|  | Over 40 | 65 | 2.728 | .729 |  |  |
| Financial risk | 20s and 30s | 66 | 3.828 | .646 | .075 | .940 |
|  | Over 40 | 65 | 3.821 | .534 |  |  |
| Workload | 20s and 30s | 66 | 3.273 | .825 | .975 | .332 |
|  | Over 40 | 65 | 3.138 | .750 |  |  |
