# Supplementary figures and images for: An Investigation of Factors Influencing the Postponement of the Use of Distributed Research Networks in South Korea: Web-Based Users’ Survey Study
Source: JMIR Form Res. 2023 Apr 12;7:e40660. doi: 10.2196/40660 (PMC10134027; doi:10.2196/40660)

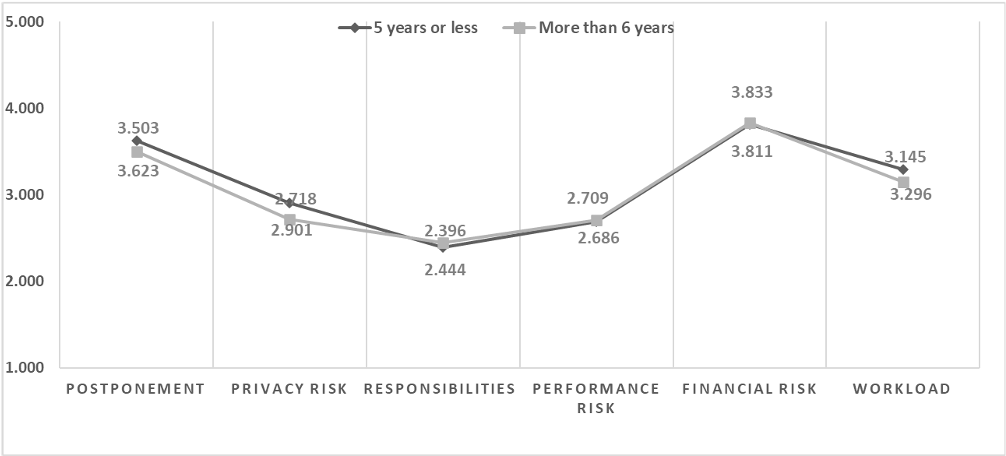

Supplement: Multimedia Appendix 2 [file formative_v7i1e40660_app2.png]

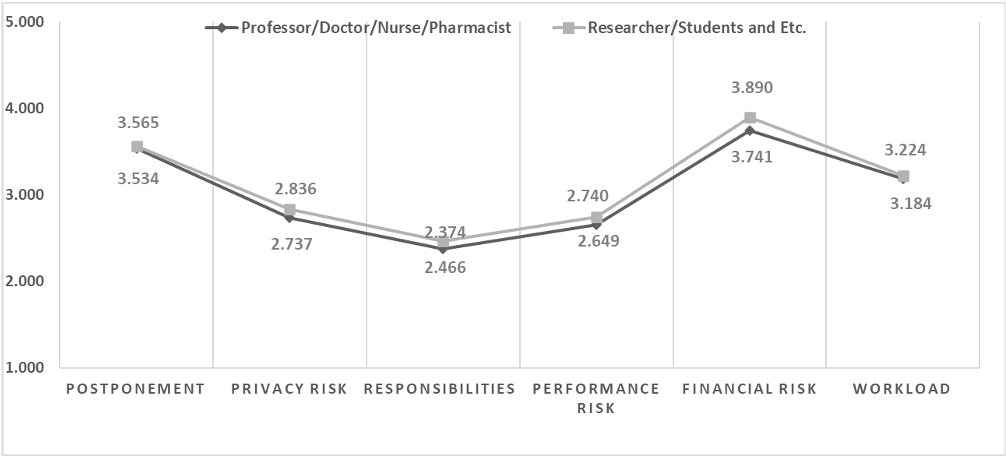

Supplement: Multimedia Appendix 3 [file formative_v7i1e40660_app3.png]

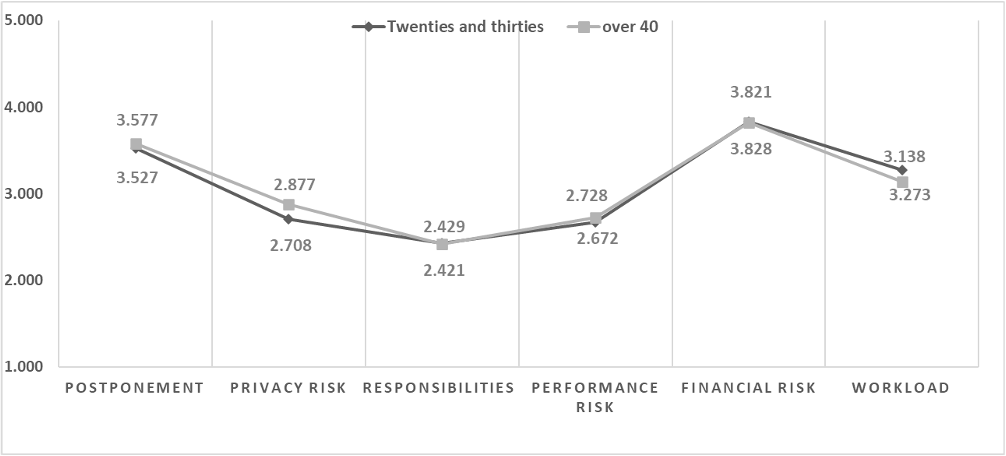

Supplement: Multimedia Appendix 4 [file formative_v7i1e40660_app4.png]
